# Intense and Superflat White Laser with 700-nm 3-dB Bandwidth and 1-mJ Pulse Energy Enabling Single-Shot Subpicosecond Pulse Laser Spectroscopy

**DOI:** 10.34133/research.0210

**Published:** 2023-08-15

**Authors:** Lihong Hong, Haiyao Yang, Liqiang Liu, Mingzhou Li, Yuanyuan Liu, Baoqin Chen, Huakang Yu, Wenbo Ju, Zhi-Yuan Li

**Affiliations:** ^1^ School of Physics and Optoelectronics, South China University of Technology, Guangzhou 510641, China.; ^2^ State Key Laboratory of Luminescent Materials and Devices, South China University of Technology, Guangzhou 510640, China.

## Abstract

An optical spectrometer is a basic spectral instrument that probes microscopic physical and chemical properties of macroscopic objects but generally suffers from difficulty in broadband time-resolved measurement. In this work, we report the creation of ultrabroadband white-light laser with a 3-dB bandwidth covering 385 to 1,080 nm, pulse energy of 1.07 mJ, and pulse duration of several hundred femtoseconds by passing 3-mJ pulse energy, 50-fs pulse duration Ti:Sapphire pulse laser through a cascaded fused silica plate and chirped periodically poled lithium niobate crystal. We utilize this unprecedented superflat, ultrabroadband, and intense femtosecond laser light source to build a single-shot (i.e., single-pulse) subpicosecond pulse laser ultraviolet–visible–near-infrared spectrometer and successfully measure various atomic and molecular absorption spectra. The single-shot ultrafast spectrometer may open up a frontier to monitor simultaneously the ultrafast dynamics of multiple physical and chemical processes in various microscopic systems.

## Introduction

Scientists use light–matter interaction to probe, monitor, understand, manipulate, and harness the microscopic physical, chemical, and biological world. An input light carrying some specific optical information (probe light) passes through a microscopic object sample, induces light–matter interaction, and transforms into output light carrying changed optical information (signal light). By measuring and analyzing such a change in carried information between probe light and signal light, one can find out the physical, chemical, and biological properties of the microscopic object. One of the simplest while powerful means toward this purpose is optical spectroscopy [1], including absorption [2–4], fluorescence [5,6], Raman scattering [7–9], infrared [10,11], spectroscopies, and many others [12,13]. All these spectroscopic methods are implemented by an instrument called spectrometers [11,14,15], which is composed of 3 crucial parts: an illumination light source with a known spectral profile, a wavelength separation device, and a photo-detector measuring the spectral intensity of signal light. By analyzing the spectral profile of signal light compared with the probe light, one can obtain the internal physical, chemical, and biological properties and their temporal evolution under external influence. This route of optical spectroscopy has played a very important role in advancing the progress of many disciplines of science [16,17]. Nonetheless, there is still demand to further push forward optical spectroscopy to an even higher level for attacking some unresolved fundamental problems in basic science.

One problem is the capability to probe simultaneously 2 or more microscopic physical, chemical, and biological processes that occur in a rather wide spectral range and monitor their temporal evolution dynamics on an ultrafast time scale. For instance, a heavy atom exhibits multiple energy levels of atomic states with complex distribution, while a molecule has an even more complicated energy level distribution profile. These atomic or molecular quantum states can have their energy levels distributed across a rather broad spectral range from ultraviolet, visible, to near-infrared (UV-Vis-NIR). When one only focuses on the transition between 2 specific quantum states and the induced internal change of atom and molecule, one can use a narrow-band ultrafast femtosecond pulse laser to excite and probe the ultrafast temporal evolution dynamics of such a change via the well-studied pump-probe technique [2,18–21]. Yet, if one would like to excite multiple transition processes among a series of energy levels and probe the induced physical and chemical change with an ultrafast temporal resolution, then an ultrabroadband and ultrafast femtosecond pulse laser must be used. So far, such a kind of optical spectroscopy technology is very much underdeveloped.

In this work, we report the creation of a bright, ultrabroadband, and superflat supercontinuum UV-Vis-NIR white-light laser and the building of a single-shot (i.e., single-pulse) subpicosecond pulse laser spectrometer. The white-light laser has an unprecedented 3-dB bandwidth of about 700 nm (ranging from 385 to 1,080 nm), pulse energy of 1.07 mJ, and pulse duration of several hundred femtoseconds. It is generated by passing 3-mJ pulse energy, 50-fs pulse duration Ti:Sapphire femtosecond laser through a cascaded fused silica plate and chirped periodically poled lithium niobate (CPPLN) crystal. A single-shot subpicosecond pulse laser spectrometer is made by interfacing this plateau-like subpicosecond white-light laser as the illumination light source with a dispersion grating and charge-coupled detector (CCD). Broadband absorbance spectra of multiple atoms or molecules are simultaneously measured by this newly constructed spectrometer. The goal is to develop a simple, powerful, and reliable ultrabroadband ultrafast spectroscopic tool for simultaneously investigating multiple microscopic physical and chemical processes of atoms, molecules, and materials and to deepen our understanding of the basic science in these microscopic systems.

## Results

### Creation of ultrabroadband subpicosecond white-light laser source

Up to date, there have been many schemes toward supercontinuum white-light laser, but most of them utilize various third-order nonlinear effects (3^rd^-NL) [self-phase modulation (SPM), 4-wave mixing, stimulated Raman scattering, etc.] of high-peak-power picosecond and femtosecond pulse laser interacting with amorphous solid materials (like silica, fluoride, and chalcogenide glass) in the form of microstructured photonic crystal fibers [22,23] or homogeneous plates [24,25], or with rare gases (He, Ar, etc.) filled within hollow-core silica fibers [26,27]. However, these purely 3^rd^-NL schemes always encounter certain limitations in the balanced performance of spectral bandwidth, spectral flatness, and pulse energy due to the tiny modal area or the dispersion properties of transport waves. Another more powerful means to expand the spectral range of the laser is various second-order nonlinear effects (2^nd^-NL) via the promising route of quasi-phase matching (QPM) scheme [28–31]. However, these existing QPM routes also have difficulty in high-quality broadband laser generation with limited spectral bandwidth, not flat enough spectral profile, and reduced conversion efficiency. Frankly speaking, it becomes a great challenge to resolve these bad limitations existing in both 2^nd^-NL and 3^rd^-NL regimes and make the best of both worlds.

**Fig. 1.**
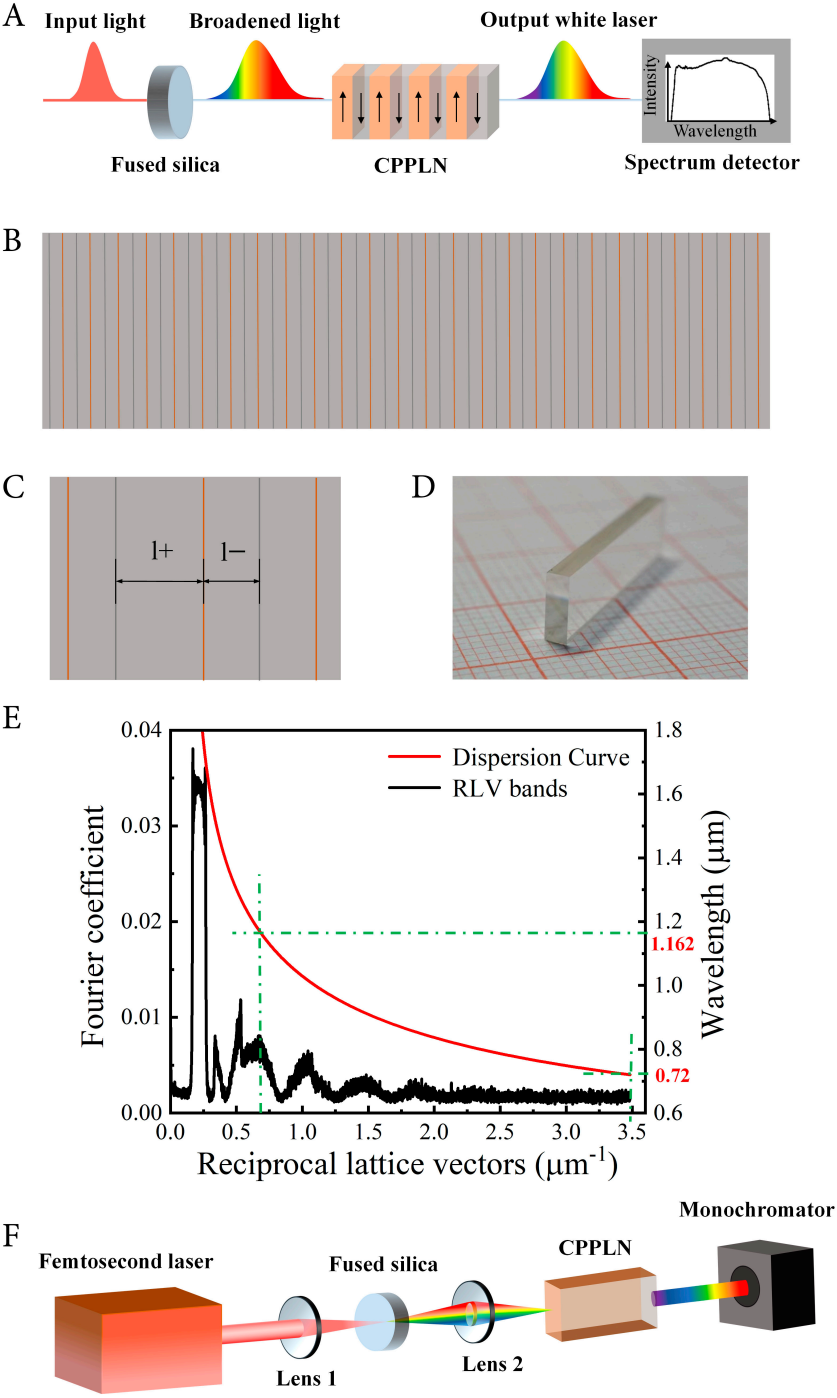
Device design of ultrabroadband white-light laser. (A) Mechanism of ultrabroadband white-light laser generation through cascaded fused silica plate and CPPLN via synergic 2^nd^-NL and 3^rd^-NL. (B) Microscopic image of the fabricated sample surface of a typical one-dimensional CPPLN structure. (C) High-magnification view of the CPPLN sample consisting of unequal negative and positive domains. (D) Fabricated CPPLN sample. (E) Phase–mismatch curve for SHG process in a lithium niobate (LiNbO_3_) crystal combined with the Fourier spectral curve for CPPLN structure in broadband SHG processes. (F) Experimental setup of ultrabroadband white-light generation from the silica-CPPLN module.

Our current scheme fully incorporates the power of 3^rd^-NL in expanding the spectral range by using simple but versatile amorphous bulk solid material (like fused silica) and the even greater power of 2^nd^-NL (much stronger in strength than 3^rd^-NL) in expanding the spectral range by using a bulk nonlinear crystal with deliberately designed QPM capabilities in an ultrabroad spectral band. In our experiment, we deliberately manipulate the overall NL response from a cascaded optical module composed of the fused silica plate and a specially designed CPPLN sample, as briefly illustrated in Fig. 1A. First, an 8-mm-thick fused silica plate is used to generate a considerably spectrum-broadened pulse signal irradiated by a high-pulse-energy Ti:Sapphire femtosecond pulse laser, where the spectral broadening mechanism is dominated by the 3^rd^-NL SPM process. This 3^rd^-NL spectrum-broadened light from bulk material provides a bright, broad, coherent, and stable pump source for the next second harmonic generation (SHG) process of CPPLN crystal. Then, the supercontinuum femtosecond laser transmits through the designed CPPLN crystal with a vast number of reciprocal lattice vector (RLV) bands to ignite continuous broadband 2^nd^-NL interactions, finally leading to the creation of ultrabroadband subpicosecond pulse white laser. Apparently, the big success of our configuration stems mainly from (a) having a high-energy-density broadband pump laser to generate a sufficiently intense power density (namely, intensity) for inducing very strong effect of 3^rd^-NL spectral broadening, but still does not cause optical damage to silica plate, and (b) a nonlinear material having ultrabroad QPM bandwidth to support high-efficiency SHG processes.

First, we make a specific design to the CPPLN sample for supporting a wide pump condition, which involves multi-order RLV bands with relatively larger effective nonlinear coefficients to motivate effective and balanced SHG interactions (see Materials and Methods for more details). The typical microscopic diagrams of the CPPLN sample are explicitly illustrated in Fig. 1A to C, where one can clearly see the asymmetric characteristic between negative and positive poled domains. The designed poling period ranges from 38 μm to 22 μm with a fixed negative domain length of 15 μm. The fabricated *z*-cut CPPLN sample with 5% MgO doped has a dimension in length, width, and thickness of 20 mm × 6 mm × 2 mm, respectively, as displayed in Fig. 1D. The RLV distributions within the CPPLN sample alongside the phase mismatching curve for SHG [against the fundamental-wave (FW) wavelength] are plotted in Fig. 1E. One can find that the SHG phase mismatching curve intersects with multiple effective RLV bands throughout an ultrabroad wavelength range, in particular the high-energy waveband of 0.72 to 1.162 μm. This indicates that such a CPPLN sample with sufficient SHG operating bandwidth should possess the ability to enable a series of continuous broadband SHG processes and finally yield the creation of a supercontinuum white-light laser.

**Fig. 2.**
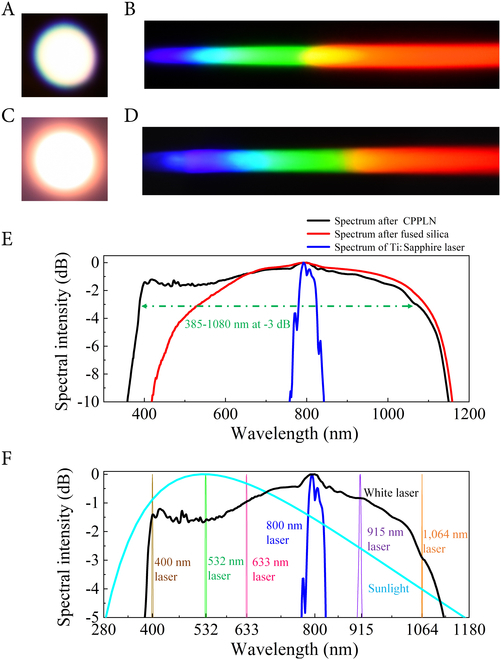
Measurement results of ultrabroadband UV-Vis-NIR white-light laser. (A) Supercontinuum spot emitting from the 8-mm-thick fused silica sample. (B) First-order diffraction beam of (A). (C) White-light spot emitting from the CPPLN sample. (D) First-order diffraction beam of (C). (E) Supercontinuum spectra (normalized against the peak intensity) emitting from fused silica, CPPLN, and the original output spectrum of Ti:Sapphire laser, respectively. (F) Spectrum of supercontinuum white laser from the cascaded silica-CPPLN in comparison with ones of the frequently used commercial lasers, involving 400-nm blue laser, 532-nm green laser, 633-nm He–Ne laser, 800-nm Ti:Sapphire femtosecond laser, 915-nm semiconductor laser, and 1,064-nm picosecond Nd:YAG laser as well as an original sunlight (originating from a 5,500 K blackbody radiation source).

A schematic diagram of the experimental device is illustrated in Fig. 1F, where a cascaded nonlinear module consisting of fused silica and CPPLN is in the architecture of being illuminated by a 3-mJ high-energy Ti:Sapphire femtosecond laser (for full details, see Materials and Methods). The corresponding supercontinuum white laser spot emitting from the fused silica is displayed in Fig. 2A. It can be found that the overall chroma appears a warm-yellow tone due to the absence of short-wavelength parts within the visible spectrum. In contrast, a very bright and cold-tone white-light beam can be acquired by sending the white laser pump beam through the CPPLN sample, as presented in Fig. 2C. This naked-eye feature preliminarily illustrates that the output spectrum generated by the CPPLN sample contains more complete and uniform visible light components. Then, to analyze quantitatively the frequency components of the white-light beams, we first use a regular grating for a qualitative assessment of these 2-stage output laser beams. Comparing Fig. 2B with Fig. 2D, clearly, the first-order SHG diffraction beam from the CPPLN sample involves continuously visible color bands varying from purple to red. Besides, the brightness of various diffraction bands looks more outstanding than the first-stage spectrum-broadened laser light from the fused silica. One can distinguish that the upconversion light is a bright and smooth supercontinuum white laser that covers the entire visible band.

Further on, we perform a more precise spectral analysis by an optical monochromator with a silicon detector to study the performance of the output white laser beam, as described in Fig. 2E. Looking closely at the red line in Fig. 2E, one can see that a typical spectrum of 3^rd^-NL broadening supercontinuum generated in fused silica is extended from the blue end of the visible to the NIR region (540 to 1,090 nm at a criterion of 3 dB). Notably, this Vis-NIR supercontinuum makes it a very ideal broadband pump source for seeding of continuous SHG process within the CPPLN sample. Afterward, a clear white laser carrying important UV generation is emitted from the CPPLN sample, as shown by the black line in Fig. 2E. As expected, a plateau-like spectrum created by the silica-CPPLN module throughout the UV to NIR region covers from 385 to 1,080 nm with a flatness better than 3 dB. In other words, this corresponds to a full-width at half-maximum (FWHM) spectral width (equivalent to the 3-dB bandwidth) of ~700 nm. As shown in Fig. 2F, such a uniform spectral distribution in this ultrabroadband and superflat UV-Vis-NIR laser source almost covers the wavelength of a series of frequently used commercial lasers, such as 400-nm blue laser, 532-nm green laser, 633-nm He–Ne laser, 800-nm Ti:Sapphire femtosecond laser, 915-nm semiconductor laser, and 1,064-nm picosecond Nd:YAG laser. Here, the bandwidth of this unprecedented supercontinuum laser is about 16 times the bandwidth of the input Ti:Sapphire femtosecond pulse laser (slightly below 45 nm) and even broader than the bandwidth of ordinary sun light (~640 nm, covering 320 to 960 nm). The superior features of ultrabroadband spectral coverage and high flatness greatly demonstrate the ability of this supercontinuum white-light laser as a laser of many different laser wavelengths. Moreover, due to the high pump intensity and high conversion efficiency of CPPLN, the power level of this UV-Vis-NIR supercontinuum laser reaches quite a high level. Concretely, the maximum output laser energy after CPPLN is 1.07 mJ per pulse with the irradiation of an engineered broadband pump condition that is fulfilled by delivering a 3-mJ per pulse Ti:Sapphire femtosecond pump laser into the fused silica plate to initiate a 1.84-mJ per pulse supercontinuum laser. Besides, we believe that when some negative effects such as absorption loss and optical damage to materials are eliminated by changing the infrastructures of the setup, or by replacing silica with other bulk materials, the power of the output laser will be even higher.

As reported in previous works, supercontinuum from photonic optical fiber can yield more than 3 octaves in the spectral range 200 to 2,500 nm, but only acquiring a pulse energy at the very low picojoule level [23]. A continuum at 0.1-TW level is obtained from 7 thin fused silica plates but has far smaller spectral bandwidth (covering 460 to 950 nm) [24]. Supercontinuum output with nearly 7 octave bands has been obtained by combining microstructure fiber and nonlinear crystal, but with a spectral range of 340 to 40,000 nm at a much lower −70-dB level bandwidth and a single pulse energy of only 0.45 μJ [32]. Apparently, the overall performance of ultrabroadband supercontinuum laser created in our scheme is better than previous reports in terms of balanced broad bandwidth, high flatness, large pulse energy, and high peak power. Meanwhile, we have modeled the 50-fs Ti:Sapphire laser pulse transmitting the glass plate based on the nonlinear Schrodinger equation (NLSE) to include the linear dispersion and 3^rd^-NL SPM effects and find the pulse duration increases to about 200 fs. We estimate that when the pulse transmits the CPPLN, the pulse duration will further increase to 300 to 500 fs. Foreseeably, such an ultrabroadband, bright, and ultrafast laser source with a 3-dB bandwidth covering 385 to 1,080 nm, pulse duration of several hundred femtoseconds, and pulse energy up to 1.07-mJ level can be widely used for ultrafast time-resolved spectroscopy to explore the detailed ultrafast dynamics of multiple physical, chemical, and biological processes simultaneously occurring in various materials.

### Building of single-shot subpicosecond pulse laser spectrometer

It is well distinguished that ultrafast spectroscopy, like femtosecond transient absorption spectroscopy, is a powerful method for investigating a wide variety of physical, chemical, and biological processes under excitation of light or other stimuli [33], especially when implemented with the powerful pump-probe technique [2,18–21]. Yet, there is still big space to advance this technique to an even higher level, especially in the part of illumination light source. First, the bandwidth of ultrafast probe laser pulse can be further expanded to cover UV-Vis-NIR or even to mid-IR regime so that they can probe as many as possible microscopic processes simultaneously. Second, the total energy and thus the average power level of a single pulse can be further elevated to a much higher level, say ~1 mJ, so that a single pulse can support sufficient energy of light, or sufficiently large numbers of photons for acquiring a reliable spectral curve with high signal-to-noise ratio (SNR). In contrast, in the contemporary pump-probe technique, the pulse energy is much smaller (well below 1 microjoule), so it is necessary to use thousands or even millions of pulses (assuming every pulse sees the same spectral change of microscopic objects, which is not always accurately true) to accumulate, average, and analyze spectroscopic data and create spectral curves with the same-level high SNR. Third, the spectral profile, in particular, the flatness, as measured by the 3-dB bandwidth (i.e., FWHM of spectral peak), can be further improved to a high level. A superflat spectral profile can greatly help to elevate the SNR of ultrafast spectroscopy in the entire large range of spectral measurement and analysis. An ordinary sunlight, which comes from a 5,500 K blackbody radiation source, has a very flat spectral profile with the 3-dB bandwidth of about 640 nm (covering 320 to 960 nm). Unfortunately, it is a continuous source and has no use in ultrafast spectroscopy. Similar case holds for ordinary halogen lamp sources. Alternatively, supercontinuum light source coming from photonic crystal fiber has a spectral range from 400 to 2,400 nm, and nanosecond, picosecond, and even femtosecond pulse duration. However, most spectral energy is concentrated around the pump laser and only a small fraction of energy is distributed in the broad spectral range of 400 to 2,400 nm. Thus, the 3-dB bandwidth is very narrow, and it cannot serve as a good illumination source for ultrafast spectroscopy. In short, it is of great value to develop a new ultrafast time-resolved spectroscopy technique based on a genuine illumination source that has simultaneously several merits as ultrashort temporal duration, extremely large bandwidth, superflat spectral profile, and large pulse energy. This new technique should shed a whole new light on ultrabroadband spectroscopic detection to resolve real-time diversiform atomic or molecular physical and chemical processes like electron transitions and molecular vibrations.

**Fig. 3.**
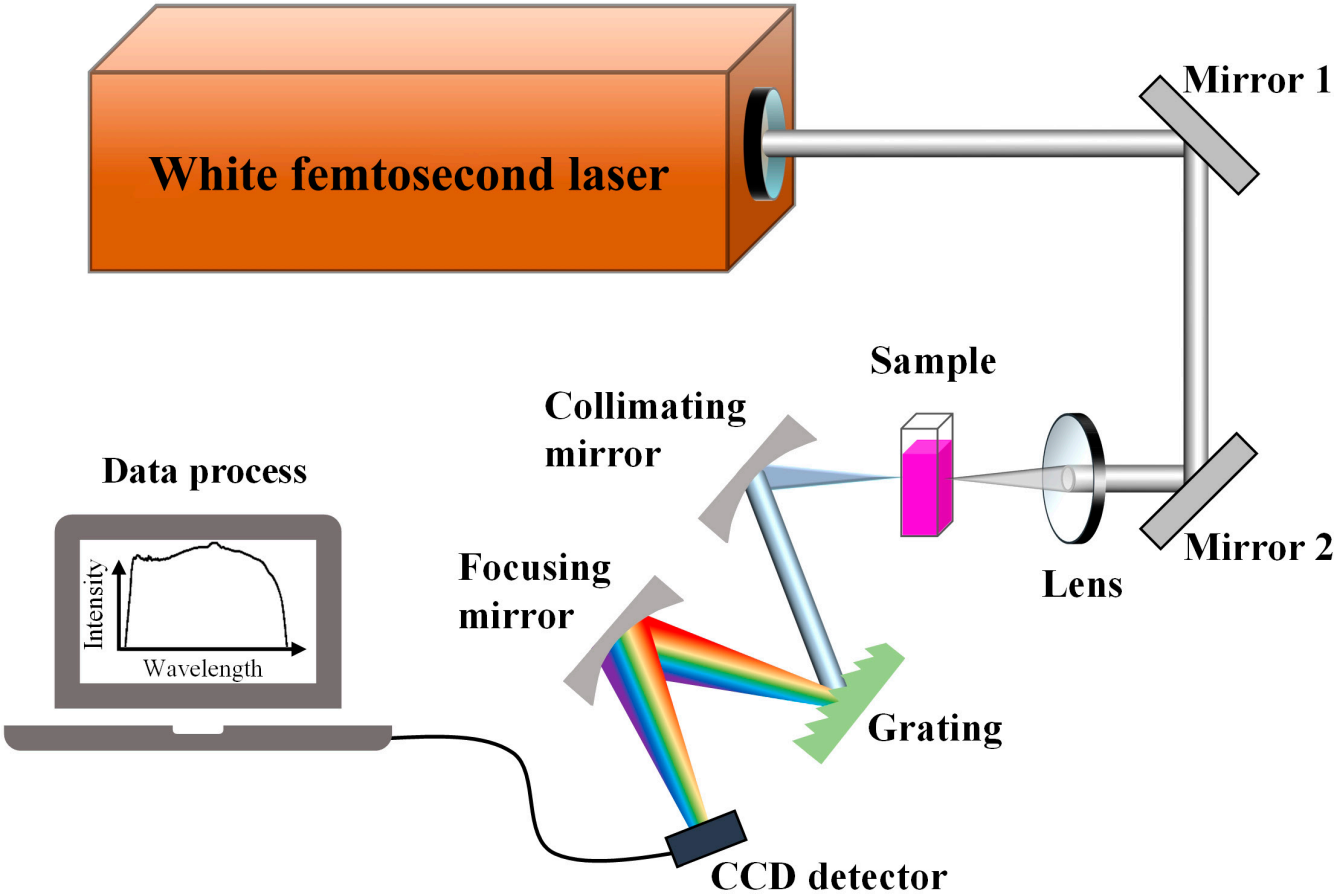
Device fabrication of the single-shot subpicosecond pulse laser spectrometer. This device consists of 3 important components: an ultrabroadband white-light femtosecond laser source for illumination, a wavelength-separated reflective diffraction grating device (600 lines/mm with blazed wavelength at 300 nm), and a CCD detector with 2,048 pixels for recording spectral intensity.

As we described above, our innovative cascaded device can successfully generate a high-pulse-energy, superflat, ultrashort, and ultrabroadband UV-Vis-NIR subpicosecond light source, which is quite applicable for broadband ultrafast spectroscopy in terms of the spectral range and flatness, optical density, and time resolution. Here, we implement a single-shot subpicosecond pulse laser spectrometer aiming to allow for long-cherished high-energy ultrabroadband UV-Vis-NIR white-light laser detection with pulse duration of several hundred femtoseconds. Figure 3 is the schematic drawing of the single-shot ultrafast spectrometer device, which is composed of the newly created ultrabroadband white-light subpicosecond laser source and a homemade spectral analysis and imaging equipment. In measurements, the white-light subpicosecond laser for a single shot is used to illuminate the samples (organic dye solutions or alkali metal gases) via a focused lens. The signal light is dispersed by a regular reflective diffraction grating with 600 lines/mm blazed at 300 nm into a line CCD detector (Hamamatsu, S11639). Finally, the transmitted spectrum at every laser shot measured on the line CCD is sent to a digital to analog converter with 16-bit resolution to proceed with spectrum construction. A full description of the system is provided in Materials and Methods.

### Atomic and molecular absorption spectroscopy

**Fig. 4.**
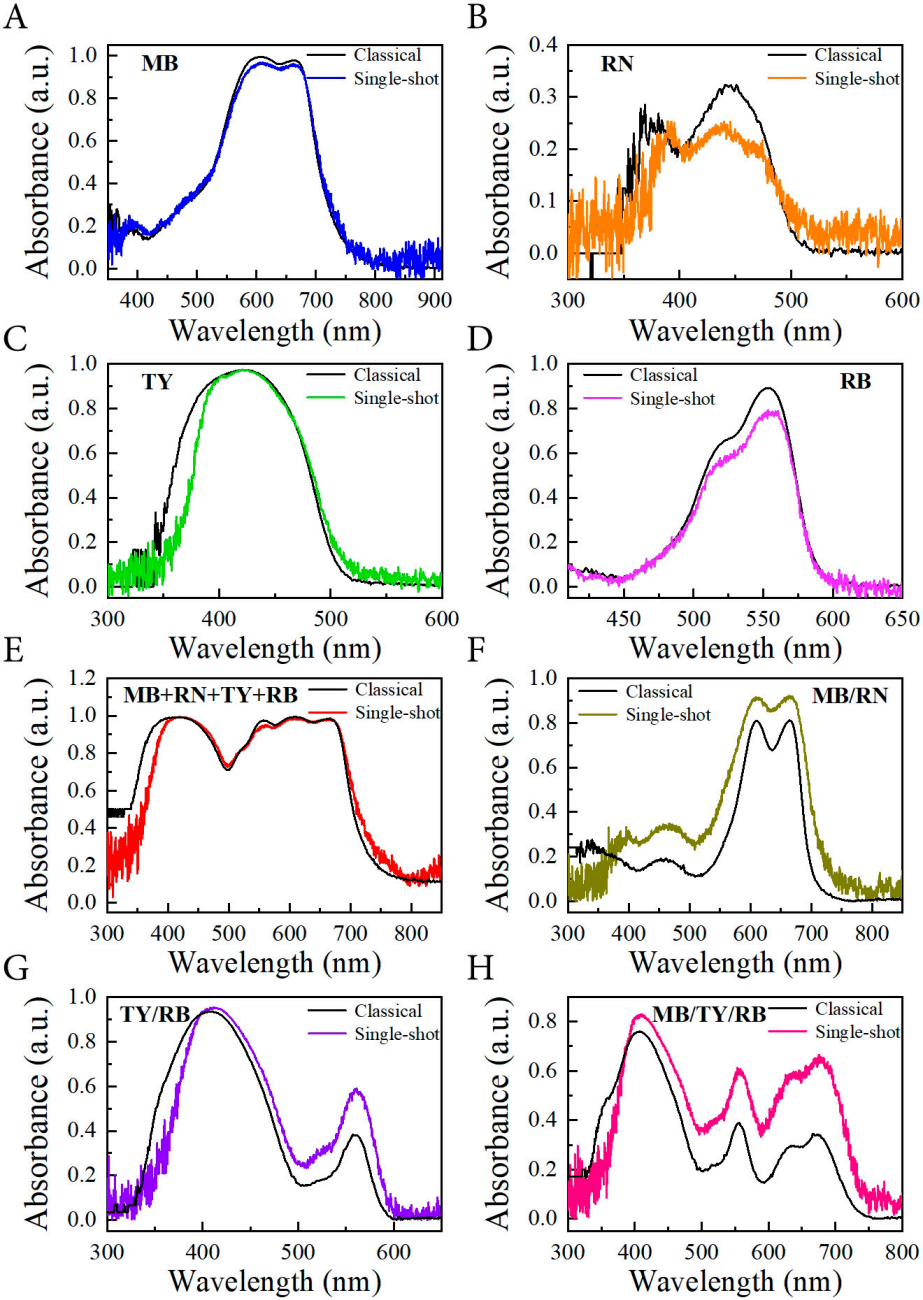
Molecular absorption spectra of methylene blue (MB), riboflavin (RN), titan yellow (TY), and rhodamine B (RB) aqueous solution as well as mixtures of multiple molecular species measured by the single-shot ultrafast spectrometer. (A) MB aqueous solution (0.5 mg/ml). (B) RN aqueous solution (1 mg/ml). (C) TY aqueous solution (0.5 mg/ml). (D) RB aqueous solution (0.5 mg/ml). Note that the samples are placed in demountable quartz cuvettes with a path length of 1 mm. (E) Side-by-side arrangement of 4 samples of (A) to (D). (F) Mixture of MB/RN in a 1-mm-path quartz cuvette with a volume ratio of 1:1. (G) Mixture of TY/RB in a 1-mm-path quartz cuvette with a volume ratio of 1:1. (H) Mixture of MB/TY/RB in a 1-mm-path quartz cuvette with a volume ratio of 1:1:1. Solid black lines in each graph were obtained by a classical spectroscopic mean combining a broadband thermal light source (Thorlabs SLS201L) with a monochromator (zolix, omni-λ 3017i) for comparison, with a recording time of 10 min under a pump power of 51 mW.

To demonstrate the spectroscopic capability of the single-shot ultrafast spectrometer system, we first carry out a molecular spectroscopic study for some organic dyes like methylene blue (MB), riboflavin (RN), titan yellow (TY), and rhodamine B (RB). We prepare the aqueous solutions of these organic dyes in demountable quartz cuvettes with a path length of 1 mm (see Materials and Methods for more details). In Fig. 4, the molecular absorption spectra, extracted by our single-shot spectrometer, are displayed with different colored lines and compared with the spectra obtained from a classical spectroscopic setup composed of a broadband halogen light source (Thorlabs SLS201L, range: 360 to 2,800 nm) combined with a commercial monochromator (zolix, omni-λ 3017i) and a point-by-point scanning operation scheme (black solid curves). Here, the datasets have been normalized by a reference spectrum of the transmitted radiation of distilled water to reproduce all the spectral features of the radiation. One can find that the absorption spectrum of each single species (shown in Fig. 4A to D) is very well reproduced in both shape and absorption maxima. Concretely, the absorption spectrum of MB in water shows its signature features of a long-wavelength maximum peak at 665 nm and a short wavelength shoulder at 608 nm among the overall absorption band of 400 to 800 nm (see the blue curve in Fig. 4A) [34]. The single spectrum of RN shows 2 maximum peaks at 392 and 445 nm, respectively, at the UV-Vis absorption band of 300 to 500 nm (see the orange curve in Fig. 4B) [35]. In the case of TY dye solution, the UV-Vis absorption spectrum spans from 300 to 500 nm with the maximum peak located at around 410 nm (see the green curve in Fig. 4C) [36]. For the RB solution, the absorption band ranges from 450 to 600 nm and displays the absorption maxima at 556 nm (see the magenta curve in Fig. 4D) [37]. Apparently, these experimental absorption bands nearly fit in both wavelength range and overall spectral shape to the reference ones obtained by a classical higher-resolution measurement (see black lines in Fig. 4A to D), implying good spectral investigation power of this new manufacturing single-shot spectrometer. Also notice that among all these spectra, only a slight deviation of the maximum absorbance peak at the shorter wavelength from the reference spectrum of RN aqueous solution exists, and this is mainly attributed to long-term exposure of the light source to the sample [35], referring to the technological defect involved within the traditional time-consuming point-by-point scanning operation scheme [19].

To further appreciate the broadband spectroscopic capability of this newly built single-shot ultrafast spectrometer, we perform additional spectral demonstrations of mixtures of multiple molecular species. At first, the above 4 samples are measured by a side-by-side arrangement. A conterminous ultrabroadband absorption band (covering 300 to 800 nm) with prominent characteristics of multiple distinct absorption maxima is unambiguously observed (see Fig. 4E), showing the capability of multi-species broadband detection of our single-shot spectrometer. Then, we acquire multiple absorption lines from several mixture solutions of MB/RN, TY/RB, and MB/TY/RB, which are mixed in equal proportions and stored in 1-mm-path quartz cuvettes, respectively. The compounded absorption spectra, as shown in Fig. 4F to H, clearly disclose the typical maximum absorbance of these mixture species (which have been displayed in Fig. 4A to D) in an extremely broad band within the UV-Vis-NIR window. Moreover, these lines are also quite consistent with the spectra obtained by the traditional method. Obviously, the single-shot spectrometer has beautifully delivered the performance of simultaneous measurement of the spectra of multiple species. Another advantage arising from this new configuration is that the time resolution is millions of times higher than the traditional measurement method, e.g., using illumination by a halogen lamp.

**Fig. 5.**
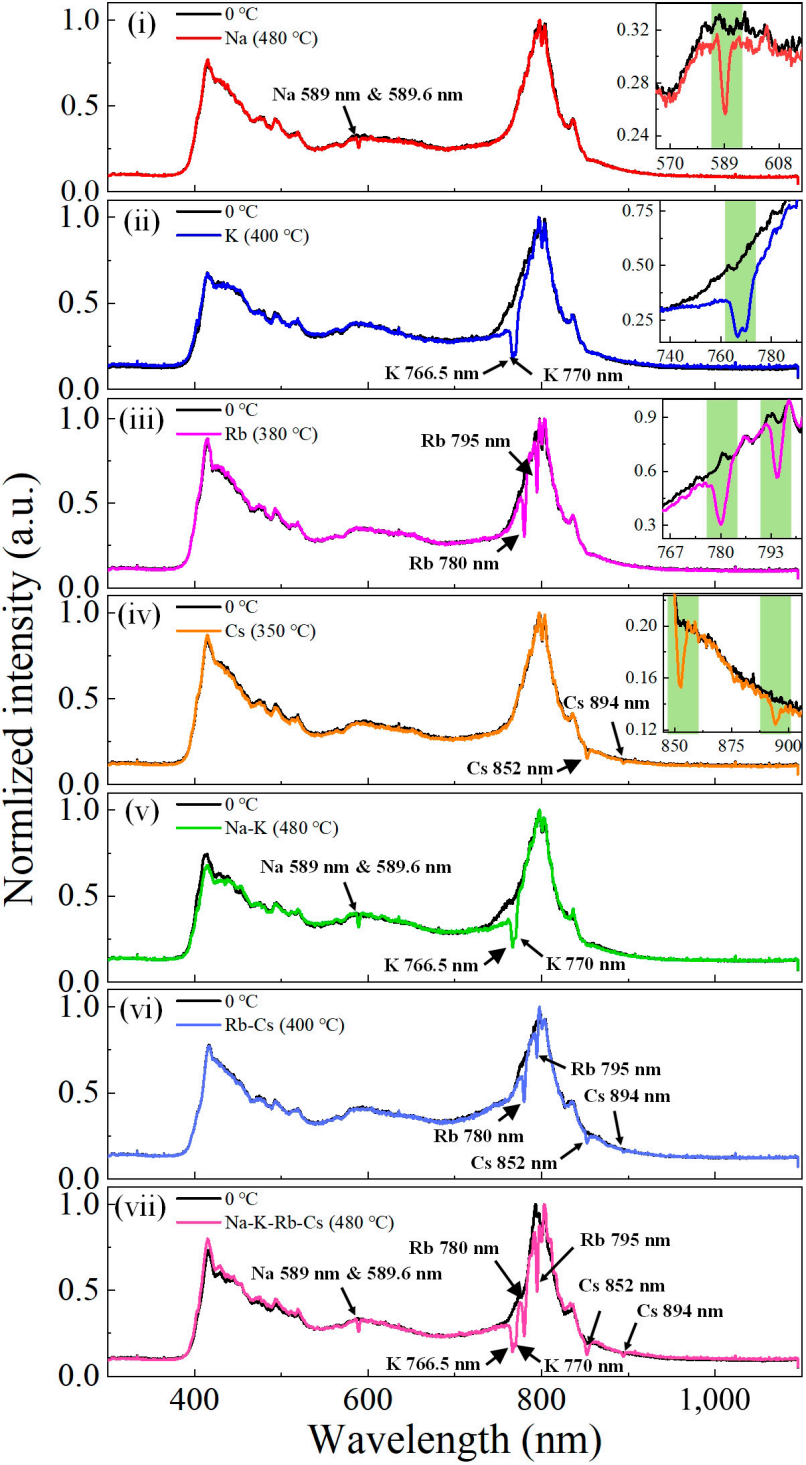
Atomic absorption spectra of 4 classic alkali metal atoms and their mixtures under different heating temperatures. (i) Na (at 480 °C), (ii) K (at 400 °C), (iii) Rb (at 380 °C), (iv) Cs (at 350 °C), (v) Na–K (at 480 °C), (vi) Rb–Cs (at 400 °C), and (vii) Na–K–Rb–Cs (at 480 °C) in argon atmosphere (measured in demountable quartz cuvettes with a path length of 15 mm), recorded by the single-shot ultrafast spectrometer. Dark black lines represent the white-light laser reference spectra of different samples under unheated conditions. The insets in (i) to (iv) are magnifications of the regions around the atomic absorption lines.

The broadband spectral-resolving power of our single-shot ultrafast spectrometer can be further evaluated by atomic spectroscopy. We prepare the samples of 4 classic alkali metal atoms and their mixtures, namely, sodium (Na), potassium (K), rubidium (Rb), cesium (Cs), Na–K, Rb–Cs, and Na–K–Rb–Cs, in demountable quartz cuvettes with the path length of 15 mm (see Materials and Methods for more details). Here, a temperature-controlled hot runner copper jacketed heater is used to heat the sample to a certain temperature to achieve a state of saturated vapor pressure for atomic spectrum measurement. In the first step, we measure the atomic absorption spectra of 4 usual alkali metals by our homemade single-shot spectrometer, as displayed in the transmission spectral curves of Fig. 5 (i to iv). For reference, the emission spectra taken from the output white laser are also shown (black lines). The absorption spectra of these alkali metal atoms at different heating temperatures encompass a sequence of sensitive absorbance lines of 589.0/589.6, 766.5/770, 780/795, and 852/894 nm, respectively, fitting well to their corresponding transition lines of energy levels [38]. These absorption lines are caused by the homogeneous metallic streams under the heating of a flat hot runner copper heater. Clearly, this single-shot spectrometer can accurately analyze a single-species atomic sample regardless of its absorbance in the visible or near-infrared region. Unfortunately, the spectrum obtained by this single-shot spectrometer cannot distinctly discriminate the absorbance lines of Na atoms at 589 and 589.6 nm due to the limited spectral resolution of our CCD detector. Subsequently, the ultrabroadband atomic-scale detection of single-shot ultrafast spectrometer is further highlighted by simultaneous measurements of multiple-species atomic absorption bands. Here, we prepare the mixtures by blending 2 or 4 alkali metals in 15-mm-thick cuvettes, respectively. One can clearly see that the mixed absorbance spectra are reconstructed with relevant single atomic signatures (see Fig. 5, i to iv). Particularly, looking closely into Fig. 5 (vii), one can distinguish that the transmittance spectrum of the mixture of Na–K–Rb–Cs involves all single atomic resonance lines of 589.0/589.6, 766.5/770, 780/795, and 852/894 nm, which are simultaneously measured and disclosed inside such an ultrabroadband spectral working range of single-shot spectrometer with subpicosecond timescale. Notice that the shift at a wavelength around 800 nm between the 2 spectra may be due to the effect of environmental perturbations on the spectral output state of the input 800-nm Ti:Sapphire femtosecond laser. Accordingly, the above results effectively reconfirm that this single-shot spectroscopic device is capable of completely sampling a large number of absorption lines for single or multiple species encompassing the extremely wide UV-Vis-NIR spectral range and thus offers a promising tool to study atomic-scale ultrafast dynamics with high temporal resolution for multiple species and multiple processes.

## Discussion

The proposed single-shot subpicosecond pulse laser spectrometer architecture has eventually been proven to allow the accurate spectral measurements of atoms and molecules, certifying excellent spectrographic capability of multiple-species detection with extremely broad spectral range and a million-fold enhanced temporal resolution, as well as higher reproducibility and reliability compared with conventional spectrometer architecture and measurement. Splendidly, the success is attributed to several factors. First, the creation of a high-energy-per-pulse ultrabroadband subpicosecond light source based on our ingenious cascaded silica-CPPLN optical module is ideally suited to ultrafast and ultrabroadband spectroscopy. Here, the practical implementation of our innovative nonlinear optical scheme provides a promising method for the construction of ultrabroadband supercontinuum subpicosecond laser source for high-precision laser spectroscopy with extremely distinctive features in terms of broad bandwidth and high flatness (~700-nm 3-dB bandwidth), large pulse energy (up to 1.07 mJ), high average and peak power, ultrashort pulse duration of several hundred femtoseconds, and high spatial and temporal coherence. These beautiful features would never be resolved by using the usual halogen lamp or optical parametric amplification (OPA) femtosecond pulse laser as the illumination light source in conventional optical spectrometers. Second, a homemade wavelength separation and detecting device is fabricated and tested to match such an ultrafast and ultrabroadband illumination mode in the single-shot ultrafast spectrometer, and the conventional point-by-point scanning scheme of spectroscopy is discarded. The achieved spectral range of detection covers from 300 to 1,100 nm with a resolution of 2.5 nm, with the signals accumulating time as short as a minimum 30 μs. One can see that this high-speed single-shot measurement effectively obliterates the obstacle of time-consuming spectral signal measurement, and laser damage accumulating on the sample existing in traditional step-scan methods and setups like monochromator combined with photo-detector. Meanwhile, the single-shot spectrometer allows to obtain spectral signals over a UV-Vis-NIR ultrabroadband range at every single measurement and is thus far superior in speed and efficiency to commercial composite grating models and instruments like commercial Horiba 330 or 550, which comprise segmentation of multiple spectral ranges.

In our previous work [31], we obtained a flat supercontinuum spectrum with a 10-dB bandwidth covering 375 to 1,200 nm and with 20-dB bandwidth covering 350 to 1,450 nm in the UV-Vis-NIR regime. However, the pulse energy is much smaller (~0.17 mJ), and the 3-dB bandwidth is very narrow (covering 700 to 860 nm, ~160 nm). At the same time, 0.5 mJ is the maximum and optimal pump laser energy that the system can withstand to support the production of a supercontinuum pump laser with a pumping bandwidth covering 700 to 1,700 nm to support ultrabroadband SHG of the designed CPPLN sample. Apparently, the supercontinuum white laser in this setup cannot serve as a good illumination source for ultrafast and ultrabroadband spectroscopy with high SNR due to the lack of several merits such as extremely large bandwidth, superflat spectral profile, and large pulse energy. In contrast, the new technique of this work relies on the further deliberate manipulation of the overall NL response from a cascaded optical module composed of a shorter 8-mm-thick fused silica plate and a specially designed CPPLN sample with continuous and broadband RLVs to make it capable of supporting higher 3-mJ Ti:Sapphire pump pulse energy. This highly adaptive configuration finally enables the creation of a supercontinuum white laser with larger pulse energy, larger bandwidth range, and higher spectral flatness. Such an ultrabroadband white laser covering UV-Vis-NIR regime with a much higher pulse energy level (~1 mJ) and a higher spectral flatness (3-dB bandwidth ~700 nm) can be extremely suitable for the single-shot ultrafast spectroscopy in the entire large range of spectral measurement and analysis with high SNR.

In conclusion, we have successfully developed a new type of single-shot subpicosecond pulse laser spectrometer by using our developed bright, coherent, ultrabroadband, and superflat subpicosecond white laser as the illumination light source, and demonstrated the ability to simultaneously probe multiple microscopic species and macroscopic samples over a broad spectral range of 300 to 1,100 nm. Meanwhile, the absorbance spectra of various atoms and molecules can be obtained with a single-shot pulse with subpicosecond temporal resolution. To the best of our knowledge, this work is the first to expand the spectral range of a spectrometer spanning from UV to NIR region (3-dB bandwidth covering 385 to 1,080 nm) together with an ultrahigh pulse energy (at >1-mJ level) and pulse duration of several hundred femtoseconds. This advancement provides a practical way to simultaneously investigate the ultrafast dynamics of multiple microscopic physical and chemical processes of atoms, molecules, and materials, which will greatly enrich the physical understanding of microcosmic systems. Additionally, the potential applications of our ultrafast and ultrabroadband single-shot spectrometer are versatile, especially for transient measurements of a large number of fast random or nonreproducible unidirectional structural changes. The expansion of the spectral range of single-shot ultrafast spectrometers is expected to have a significant impact on the development of sensor products, clinical treatment equipment, scientific instruments, etc. Furthermore, when the spectroscopic specification of our system is combined with a faster detector, higher-spectral-resolution grating-optic equipment, and pulse compression technology, it can greatly enhance optical sensitivity, spectral resolution, temporal resolution, and SNR of ultrafast spectroscopy to fit specific applications in physics, chemistry, materials science, and biology. We envisage that our ultrabroadband high-temporal-resolution single-shot spectrometer can be an essential component for applications in basic science areas like atomic, molecular, and optical physics, condensed matter physics, analytical chemistry, physical chemistry, molecular and cell biology, material science, and high-tech areas like electronics, optoelectronics, quantum communication, environmental monitoring, medical diagnosis, frontier spectroscopic imaging, and so on.

## Materials and Methods

### Principle of CPPLN design

For the principle of CPPLN design, an effective nonlinear coefficient model is adopted to make a qualitative analysis of designed CPPLN sample in terms of the availability of QPM and energy conversion efficiency [39,40]. The poling period of the CPPLN crystal is designed to follow the formula Λ(*y*) = Λ_0_/[1 + (*D*_g_Λ_0_*y*/2*π*)] along the propagation direction of the pump laser (denoted as +*y*), where *D*_g_ is the chirp date. In our experiment, the designed lattice pitch size reduces from 38 to 22 μm with the starting period Λ_0_ = 38 μm, chirp rate *D*_g_ = 6 μm^−2^, and a fixed width of negative domain at 15 μm. The sample has a total length of about 20 mm. The CPPLN samples with 5% MgO doped can be prepared via the standard electric poling technique [28–30,39,40]. A series of successive broadband QPM bands are provided to match the high-energy-per-pulse supercontinuum pump wavelength (0.72 to 1.162 μm) as described in Fig. 1E Here, the higher-order RLV bands with lower effective nonlinear coefficients are deliberately designed to contribute to the SHG process of the short-wavelength pump laser (around 720 to 900 nm). This is mainly due to the fact that the incident supercontinuum laser pulse produced by silica is much more intense in the short wavelength part than in the long-wavelength part. Such a unique scheme will allow for efficient and balanced SHG from the CPPLN sample via a series of cascaded broadband QPM processes and finally ensure a dazzling and flat white laser output toward the UV-Vis-NIR region (385 to 1,080 nm at 3 dB).

### Experiment of white-light laser source

In the experiment, we used a fused silica plate sample with a dimension of 25 mm × 8 mm to excite the first coherent supercontinuum. The input pump Ti:Sapphire femtosecond laser (Coherent, Astrella USP) emitted pulses of duration 50 fs at center wavelength 780 nm with 1-kHz repetition rate, corresponding to a pulse FWHM bandwidth of about 45 nm, a setting laser average power of 3 W, and an energy per pulse of 3 mJ. Notice that the repetition rate could be freely adjusted down to 1 Hz (one shot per second) or straightly set to a nonrepeating single-shot (i.e., single-pulse) mode for the sake of serving as the illumination light source of the single-shot subpicosecond pulse laser spectrometer. The Ti:Sapphire laser beam was focused into the 8-mm-thick fused silica through an *f* = 100 mm fused silica lens so that a beam in diameter of around 4 mm at the point of incidence transmits through the fused silica plate to initiate a dramatic spectral broadening. Meanwhile, it is possible to dramatically regulate the output spectrum profile and energy power from the same silica plate with the optimization for the focusing conditions. The output beam profile and energy power of the 3^rd^-NL spectrum-broadened light were monitored to firmly fit the next 2^nd^-NL part of the CPPLN crystal and avoid optical damage. Then, the ultrabroadband white-light laser was produced by focusing the fundamental-wave supercontinuum laser on the polished front surface of the *z*-cut CPPLN by an *f* = 30 mm fused silica lens with a focus spot size of around 2 mm to ignite SHG. Here, the peak intensity of the input laser beam is about 318.30 GW/cm^2^, well below the optical damage threshold for the CPPLN sample. Finally, the optical monochromator and power meter were used to make an accurate spectral analysis over the output white laser beam.

### Device fabrication of single-shot spectrometer

A full description of our single-shot ultrafast spectrometer is shown in Fig. 3. A white-light laser with an unprecedented 3-dB bandwidth of about 700 nm (ranging from 385 to 1,080 nm), a pulse energy of 1.07 mJ, and pulse duration of several hundred femtoseconds was used as an ultrabroadband illumination light source. A single-shot ultrabroadband white laser pulse, after reflection by 2 quartz mirrors coated with UV aluminum film, went through the atomic or molecular samples by a focused lens (*f* = 40 mm) and was detected by an improvised spectral acquisition system. For the broadband design, we used a small grating pitch (1.6 μm) and fundamental +1^st^-order diffraction mode to minimize the diffraction order mixing. Here, the probe-signal detection system consists of several essential elements: (a) a concave mirror with *f* = 10 mm to collimate the transmitted light into (b) a regular reflective diffraction grating (600 lines/mm, with blazed wavelength at 300 nm), (c) another concave mirror with *f* = 10 mm (both concave mirrors are coated with highly reflective coatings in the range of 300 to 1,100 nm) to focus the diffraction signal into (d) a line CCD detector (Hamamatsu, S11639), whose pixel size is 14 × 200 μm^2^ having 2,048 pixels (image reading effective photosensitive area length is 28.672 mm) and spectral response range is from 300 to 1,100 nm with a resolution of about 2.5 nm. Then, the data collected on the line CCD were sent to (e) a digital to analog converter with 16-bit resolution. Finally, the transmission spectra of signal lasers at every single shot were measured with a signal accumulating time down to a minimum 30 μs.

### Molecular sample preparation

The sample preparation of MB, RN, TY, RB, and their mixtures was as follows. First, the MB aqueous solution was prepared by mixing 5 mg of MB in 10 ml of distilled water, corresponding to the concentration of 0.5 mg/ml. Analogously, 1 mg/ml RN aqueous solution and 0.5 mg/ml TY and RB aqueous solutions were prepared. The aqueous solutions were packed into the quartz cuvettes with dimensions of 12.5 mm (length) × 3.5 mm (width) × 45 mm (height) to form a uniform optical path of 1 mm. Afterward, the equal-proportion mixtures of MB (0.5 mg/ml) and RN (1 mg/ml), TY (0.5 mg/ml) and RB (0.5 mg/ml), and MB (0.5 mg/ml), TY (0.5 mg/ml), and RB (0.5 mg/ml) were prepared in three 1-mm-path quartz cuvettes. The absorption spectra of the dyes were recorded at room temperature of 24 °C.

### Atomic sample preparation and heating equipment

Four classic alkali metal atoms and their mixtures, namely, sodium (Na), potassium (K), rubidium (Rb), cesium (Cs), Na–K, Rb–Cs, and Na–K–Rb–Cs, were prepared as follows. First, 200 mg each of Na, K, Rb, and Cs was placed into a 15-mm-thick quartz cuvette filled with argon atmosphere. As for the mixtures of Na and K, Rb and Cs, and Na, K, Rb, and Cs, 100 mg each of Na, K, Rb, and Cs was mixed into a 15-mm-thick quartz cuvette filled with argon atmosphere, respectively. In our experiment, we used a simply equipped temperature-controlled hot runner copper jacketed heater to heat the sample to a certain temperature and achieve a state of saturated vapor pressure for the measurement of atomic absorption spectra.

## Data Availability

The data that support the finding of this study are available from the corresponding author upon reasonable request.
